# Finding the Pareto front for high-entropy-alloy catalysts

**DOI:** 10.1039/d5sc06100h

**Published:** 2026-01-09

**Authors:** Chengyi Zhang, Ruihu Lu, Qi Sun, Yu Mao, Tilo Söhnel, Yan Zhao, Donald G. Truhlar, Ziyun Wang

**Affiliations:** a School of Chemical Sciences, University of Auckland Auckland 1010 New Zealand ziyun.wang@auckland.ac.nz; b College of Material Science and Engineering, Sichuan University Chengdu 610065 China yan60@hotmail.com; c Department of Chemistry and Supercomputing Institute, University of Minnesota 207 Pleasant Street S. E. Minneapolis MN 55455-0431 USA truhlar@umn.edu

## Abstract

Finding catalysts that have both high activity and high stability presents a long-standing challenge. Since optimizing activity and stability are conflicting objectives, the best one can do is find the Pareto front that yields optimal tradeoffs between these features. On the Pareto front, there is a trade-off where a portion of catalytic activity must be sacrificed to gain further stability and *vice versa*. Here, we provide a method to optimize the front by designing a multi-objective genetic algorithm that combines machine learning, graph neural network calculations, and density functional calculations. The application considered is the oxygen evolution reaction catalyzed by high-entropy alloys. We find that the Pareto front generally contains alloys with diverse elements, but that enhancing stability inevitably inflicts a toll on activity. We compare the general conclusions of our work to a survey of 545 experiments.

## Abstract 2

We provide a new genetic algorithm approach to bi-objective optimization of catalysts composed of high-entropy alloys, in which activity and stability are conflicting targets.

## Graphical abstract

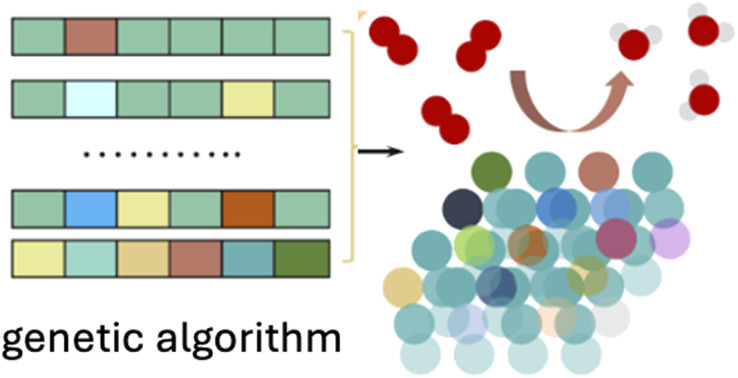

## Introduction

Improving catalytic processes remains a persistent challenge in chemistry.^1–4^ One desires a good catalyst to have robust activity and prolonged operational durability, especially for functioning under harsh conditions.^5–8^ Striking a balance between activity and stability is a foundational problem in catalysis,^9–16^ and optimizing this balance is challenging. While many catalysts possessing both good activity and good stability have been developed,^17–26^ one is usually unsure whether the trade-off between activity and stability has been optimized, and a better way to manage this trade-off would be beneficial.^27–29^

**Fig. 1:**
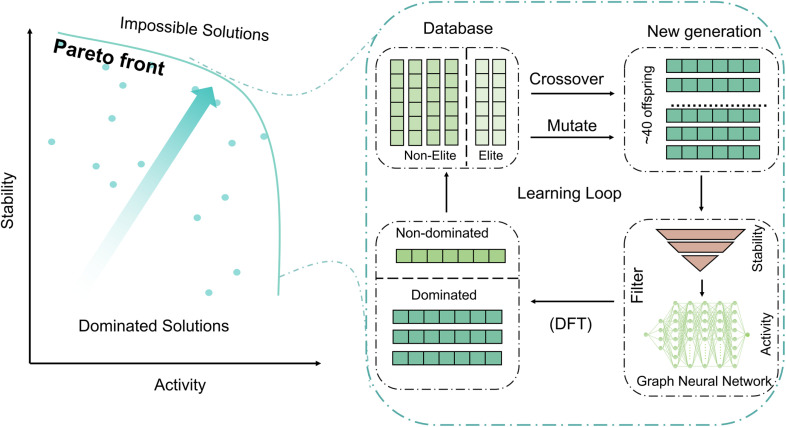
Workflow of the genetic algorithm. (left) A possible Pareto front for a bi-objective optimization of activity and stability for the oxygen evolution reaction. (right) Workflow integrating a genetic algorithm and machine learning, either with a graph neural network (in the surrogate stage) or GNN for the pre-optimization and DF theory to get the final energy (in the final stage).

A key feature of multi-objective optimization of conflicting features is that there is no optimal solution. The best one can do is find a Pareto-optimal catalyst in which one objective cannot be improved without worsening another.^30–32^ Pareto-optimized solutions to the bi-objective optimization problem are said to be non-dominated, and the set of non-dominated solutions constitutes the Pareto front. Fig. 1 illustrates that for each solution on the front, no improvement can be made in one objective without sacrificing the other. Catalysts on the Pareto front are non-inferior to each other and superior to catalysts not on the front. The present article presents a strategy for finding the Pareto front.

Key issues in multi-objective genetic algorithms are advancing the population toward the Pareto front and maintaining the diversity of the current best estimate of the Pareto front. The present algorithm combines genetic algorithms with a machine learning model and density functional calculations to advance the front, and it applies fitness sharing and niching to promote sampling the whole Pareto front, in contrast to converging to a single member of the front. As our illustrative application, we consider high-entropy alloys^33–41^ (HEAs) as catalysts for the oxygen evolution reaction (OER). This is an especially challenging problem because HEAs have a large number of possible compositions. Our work uses global optimization as a tool to uncover the fundamental activity–stability trade-off under realistic OER conditions, directly linking site-resolved OER potential barriers and Pourbaix-governed decomposition thermodynamics to experimentally observed catalyst degradation and overpotential.^42–45^

Descriptor-based methods have been widely used to predict catalyst stability and activity,^43–49^ and we formulate the present bi-objective optimization as a maximization of stability and activity descriptors. We adopt the negative of the decomposition energy per atom derived from Pourbaix analysis at 1.23 V *vs.* RHE and pH 7 as the stability descriptor because it directly reflects the electrochemical environment relevant to OER operation. For activity, we explicitly calculate the Gibbs free energies of *OH, *O, and *OOH at each adsorption site to construct the complete four-step OER free-energy profile. The overpotential is determined from the largest free-energy change minus 1.23 eV, and its negative value is adopted as the activity objective in the multi-objective optimization. With this definition, a larger (less negative) value of the descriptor corresponds to higher catalytic activity.

We consider alloys composed of Ag, Au, Cu, Ir, Ni, Pd, Pt, and Rh or a subset of these elements in a surrogate model. In our model, each catalyst structure contains 32 atoms.

The success of a genetic algorithm is determined to a large extent by the choice of its hyperparameters. Therefore, we used a two-stage procedure in which the first stage optimizes hyperparameters, and the second stage finds the Pareto front. For the second stage, we employed a graph neural network (GNN, specifically the UMA-m-1p) for pre-optimization and density functional (DF) calculations to derive the descriptors utilized by the genetic algorithm. For the first stage, we the GNN as a surrogate model to optimize the hyperparameters. The usage of a GNN in the surrogate stage allowed us to explore a broad hyperparameter space at a lower computational cost than using DF calculations. Except for the shift from GNN to GNN-DF, the two stages use the same procedures; however, the GNN stage runs the genetic algorithm many times with different choices of hyperparameters, whereas the GNN-DF stage is carried out only once (with the optimized hyperparameters) in order to find the final Pareto front.

We start each genetic algorithm run with pure face-centered-cubic (FCC) metals. This is the first generation. For the stability descriptor, we compute decomposition energy per atom; the formula is in Section S3.2 (sections and figures with a prefix S are in the SI). Before calculating the activity descriptor, we use *CatKit* to cleave surfaces and enumerate all possible hollow adsorption sites. We then calculate the adsorption energy of *O, *OH, and *OOH on each site; the activity formula is in Section S3.1.

Starting with the second generation, the stability calculation is preceded by a stability filter, which is an empirical equation to eliminate unreasonable alloy structures; the details of the filter are in Section S5. The filter is not needed in the first generation since we do not have alloys yet.

After stability fitting and activity screening, we calculate the two descriptors (by GNN in the surrogate runs and by DF in the final run), and the descriptors are used to calculate the fitness. Details of the fitness calculation are in Section S4.1. Based on the descriptors, the alloys are divided into an elite group, in which no catalyst of this generation outperforms the alloy in both activity and stability, and a non-elite group, in which at least one property, either stability or activity, is inferior to other catalysts. The elite group of the first generation forms the initial best estimate of the Pareto front. We breed successive generations by crossover and mutation, as detailed in Section S4.2. The elite group of each new generation is mixed with the Pareto front of the previous generation to obtain a new Pareto front.

We breed subsequent generations based on the population of the new Pareto front and the latest generation. Breeding is achieved through mutation and crossover. During the mutation process, a specified number of atoms are randomly selected and replaced with different metal species. The mutation number thus determines the extent of compositional diversity introduced in each generation. Breeding priority is also affected by a similarity-based selection parameter called the niche size, which is denoted as *σ*_share_. The niche size penalizes candidates that are too similar to others in the population, reducing their likelihood of similar catalysts being selected to generate offspring. This mechanism encourages exploration of a broader and more diverse region of design space.

We continue making new generations until the Pareto front converges.

As mentioned above, the surrogate stage consists of many runs with different values of the hyperparameters. As an example, consider the optimization of the number of offspring per generation, denoted as O, and the number of catalysts in each generation, denoted as G; these two hyperparameters were optimized as a pair. To do this, we carried out six independent genetic algorithm runs in each of which we fixed the total number of structures evaluated at 600, which is the product of O and G. Each pair O–G pair represents a different trade-off between breadth (more offspring per generation) and exploration depth (more generations). We obtain a different Pareto front for each O–G pair. We then combine these Pareto fronts into a global Pareto front that contains the best overall candidates. The Pareto front generated with G = 15 and O = 40 contributed the most points to the combined Pareto front, and therefore, this pair is considered the most effective and was selected as our optimized pair. More details of this optimization as given in Section S4.3.2.

The same procedure was also applied to optimize the mutation number and the niche size. Details of these runs are given in Section S4.3.3. A mutation number of 6, and a *σ*_share_ of 20% were adopted for the DF run.

All steps are summarized in Fig. 1 and in more detail in Fig. S1.

## Results & discussion

**Fig. 2:**
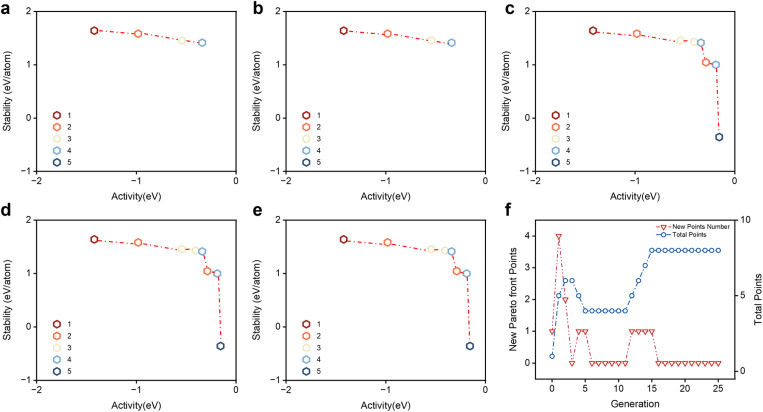
The boundary evolution by density functional calculations. The performance distribution of the alloys in individual generations (a) 1, (b) 5, (c) 10, (d) 15, and (e) 20. We utilize the transition from red to blue to signify an increase in the number of elements in the alloy. Each generation's new Pareto points and the total points on the Pareto front are shown in (f).

The final Pareto front is based solely on DF results but is obtained with hyperparameters optimized with the GNN model. Fig. 2a–e shows the evolution of the boundary as the generation number increases. We can see that by the 15th generation, trade-offs appear and settle down to a well-delineated Pareto front.

The Pareto front in Fig. 2e illustrates the trade-off between activity and stability in catalysts for the oxygen evolution reaction. In the initial optimization stages, activity and stability increased; however, after a critical point, the boost in activity was met with a corresponding decline in stability. The Pareto front's shape shows that as activity continuously increases on the front, achieving the same level of improvement in stability requires an increasingly significant sacrifice in stability.

Fig. 2f shows a converging trend of the Pareto front with the increasing generations. The Pareto front stabilizes at the 15th generation and maintains its shape for approximately ten generations, indicating convergence.

To gain insight into the relationship between activity and stability in catalysts for the oxygen evolution reaction, we compiled a dataset consisting of 545 experimental data points. For analyzing experiments, we adopted the reciprocal of the experimental overpotential to represent activity; a higher reciprocal implies lower overpotential and better activity. Adopting a measure of experimental stability is complicated by the fact that the definition of stability varies across different publications; for example, some report stability based on the time it takes for activity to decay to 90%, while others report the time it takes for activity to decay to 80%. To standardize, we choose the 90% time. The next complication is that different degradation mechanisms can lead to variations in estimating the 90% decay time. To account for this, we analyzed the activity data with three possible decay models having decay functions that vary as 
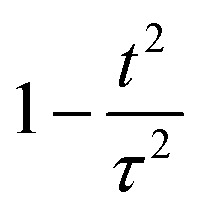
, 1 − *t*/*τ*, and e^−*t*/*τ*^, where *τ* is a fitting parameter. For each catalyst, the reported operation time and percentage of activity decay were fit to these three functions to derive the 90%-activity-decay time, which is taken as the stability descriptor. Details of the experimental data are presented in a spreadsheet file in the SI.

**Fig. 3:**
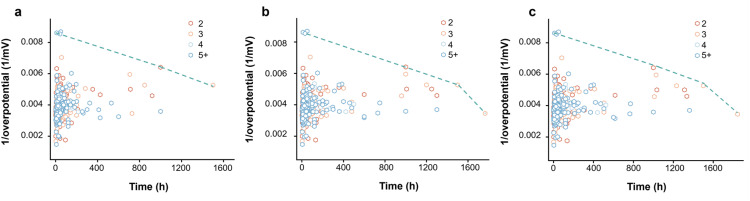
Pareto frontier from experiments. Experimental results based on concave (a), linear (b), and convex (c) fitting to estimate catalyst stability. We used the time to 90% efficiency as an indicator to determine the stability of the material.

Fig. 3a–c shows stability (90%-activity decay time) and activity (reciprocal of overpotential) for each of the three possible decay functions. In each graph, the points farther from the origin represent better-performing materials. We found that, regardless of the mathematical model used to describe activity over time, a clear and consistent trade-off between catalytic activity and stability emerges in experimental studies. This trade-off is reflected in the downward trend of the reciprocal of overpotential overtime for the best catalysts. This inherent trade-off reflects a fundamental challenge in designing catalysts, particularly for applications that require prolonged operation, such as industrial electrocatalysis and energy conversion technologies.

**Fig. 4:**
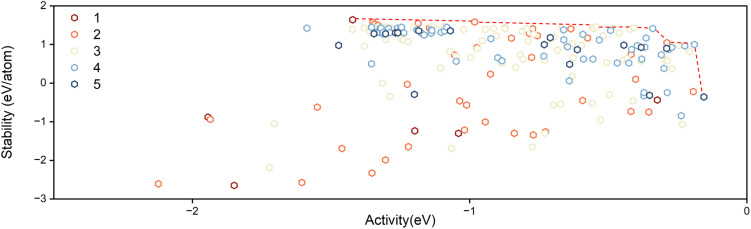
The distribution of all alloys by density functional calculations. The colours indicate the number of elements in the HEA.

We return to the DF computations in Fig. 4. By incorporating degradation thermodynamics under realistic electrochemical conditions, our framework establishes a composition-dependent Pareto frontier that reveals how HEAs strike a balance between activity and stability during OER. The results indicate that within the complex HEA compositional space, certain alloys can remain both stable and catalytically active under reaction conditions, while most conventional transition-metal surfaces readily oxidize or dissolve. This finding suggests a possible design route for future OER catalysts, namely the construction of inert metallic backbones (*e.g.*, Ag or Au) doped with a limited fraction of active transition metals (such as Cu or Pd) to generate suitable OER sites while maintaining structural integrity. Although achieving such atomic-level site control experimentally remains challenging, especially in multi-component alloys, the Pareto frontier offers a quantitative framework for understanding how stability and activity emerge collectively from composition.

Furthermore, the Pareto frontier also holds important implications for the understanding of OER catalysts based on layered double hydroxides (LDHs). In most practical systems, high-entropy or multimetallic precursors do not remain metallic but rather reconstruct *in situ* into multi-cation oxyhydroxide or LDH phases that constitute the real active state. However, the cation composition of these LDHs largely inherits that of their metallic precursors, with only minor surface enrichment or depletion observed experimentally. *Operando* X-ray studies by Dionigi *et al.* demonstrated that Ni–Fe and Co–Fe alloys rapidly transform under OER conditions into MFe(oxy)hydroxide phases, indicating that the metallic precursor primarily serves as a chemical reservoir and structural template for the active phase, indicating the close contact between the alloy precursor and the LDH.^50^

In our framework, catalytic activity is quantified using the theoretical overpotential at 1.23 V *vs.* RHE, obtained from DF calculations of the adsorption energies of *O, *OH, and *OOH intermediates following the standard four-step OER mechanism. These adsorption energies reflect each alloy's average oxophilicity, corresponding to the intrinsic tendency of its constituent elements to form M–O bonds. Numerous studies have demonstrated a linear relationship between oxygen adsorption energies on transition metals and those on their corresponding oxides, indicating that the same underlying M–O interaction governs both chemisorption and oxidation^51,52^ Consequently, the Pareto frontier established for metallic systems can be mapped onto LDHs, consistent with our results, which show that both metals and LDHs align along a shared frontier of activity and stability.

Complementing this descriptor, the decomposition energy from Pourbaix analysis at 1.23 V *vs.* RHE and pH = 7 captures the thermodynamic driving force for oxidation or dissolution. Numerous studies also demonstrate that the strength of the M–O interaction directly correlates with surface chemistry and bulk stability.^53–55^ A stronger oxygen adsorption energy reflects the formation of a stronger M–O bond, which in turn stabilizes the corresponding oxide phase and enhances the thermodynamic driving force for oxidation. In this context, a strong decomposition energy indicates excessive oxophilicity and a tendency toward rapid oxidation and structural collapse, whereas a positive value represents excessive inertness, suppressing surface reconstruction and resulting in low site density. Between these two extremes lies a narrow stability corridor where the alloy is sufficiently oxophilic to evolve into an active LDH-like surface under reaction conditions yet remains stable enough to avoid self-destruction. This delicate balance between reactivity and structural resilience defines the optimal region on the Pareto frontier, unifying the behaviour of metals and LDHs under electrochemical environments.

Our theoretical predictions agree with experimental observations. Within the Pareto frontier obtained from our simulations, alloys enriched with Au and Ag occupy the most favourable region, indicating that during iterative optimization, the system naturally tends to adopt inert noble metals as the structural backbone to preserve overall stability, while incorporating only a limited fraction of active transition metals (such as Cu or Pd) to generate OER-active sites. This compositional bias reflects the fundamental balance between activity and durability encoded in the frontier itself, namely that inert metals maintain the framework, while moderately oxophilic dopants provide the necessary binding strength for oxygen intermediates.

In current experimental systems, the primary active centers are derived from transition-metal cations such as Ni, Co, and Fe, which govern the lattice–oxygen-mediated OER pathway through redox transitions between M^2+^/M^3+^ states.^56^ However, their high oxophilicity also renders them prone to oxidation and dissolution, leading to the progressive collapse of the layered structure during operation.^57,58^ Incorporating more inert or noble elements, such as Au, Zn, V, or Cr, does not necessarily introduce new active sites; however, it can stabilize the lattice and maintain the structural framework during long-term operation. For instance, single atoms or small clusters of Au can suppress Fe leaching and confine lattice oxygen activation, thereby extending the lifetime without sacrificing activity.^59,60^ Similarly, V or Zn doping tunes the metal–oxygen covalency, promoting electronic delocalization that mitigates overoxidation and facilitates reversible redox cycles.^61,62^ These experimentally observed stabilization effects align closely with our theoretical Pareto frontier, which captures the same trade-off between activity and structural resilience across both metallic and hydroxide catalysts.

Recent *operando* investigations have provided evidence supporting this principle. Xia demonstrated that by rationally pre-designing the precursor composition, one can steer the *in situ* reconstruction pathway to form a stable and active phase under high-current conditions.^63^ Our computational Pareto framework, which simultaneously ensures sufficient oxophilicity for activation and adequate structural robustness for durability, echoes the experimentally validated concept of “precatalyst programming”. Together, these insights highlight a converging route for theory-guided synthesis, using descriptor-based Pareto mapping to pre-program alloy or precursor compositions that autonomously evolve into resilient, high-performance catalysts under realistic electrochemical environments.

## Conclusion

Catalyst optimization is crucial in chemical synthesis for achieving objectives in energy sustainability, materials science, medicinal chemistry, and environmental protection. Optimal design of catalysts is a bi-objective optimization in which activity and stability are conflicting targets. Since no catalyst will provide the best solution for both objectives, the best one can do is find a set of equally good solutions called the Pareto front or the Pareto-optimal solutions. On the front, one objective cannot be improved without worsening the other. Here, we provide a new genetic algorithm approach to the problem, and we apply it to the Pareto front of catalysts composed of high-entropy alloys, which offer exciting but challenging new opportunities for catalyst tailoring because of their vast number of possible compositions.

The bi-objective optimization of catalytic activity and stability is crucial to catalyst design. Our algorithm has five key features: (1) alloy offspring production; (2) filtering and screening; (3) hyperparameter determination in preliminary runs with more affordable calculations; (4) fitness computation by density functional calculations; (5) enrichment of the non-dominated offsprings database by a genetic algorithm that uses niche recognition to promote finding the whole Pareto front. The success of the algorithm is demonstrated by application to the difficult problem of HEA catalysts.

The relevance of a multi-objective optimization to experiment is determined in large part by the choice of features used as objectives. Our activity feature is based on explicitly resolving the full four-electron OER free-energy landscape at each surface site. For every adsorption site on every candidate HEA, we compute the Gibbs free energies of *OH, *O, and *OOH under the relevant pH and potential, and extract the potential-limiting step and corresponding overpotential by subtracting the 1.23 V equilibrium potential. This site-resolved OER overpotential is then used as the activity objective. As a result, our activity axis is not a surrogate based on a single descriptor or an empirical model but rather is the actual OER overpotential derived from the complete *OH/*O/*OOH free-energy sequence on heterogeneous HEA surfaces. Our stability feature aims to capture the *operando* electrochemical stability of HEAs under harsh OER conditions, where dissolution and phase decomposition inevitably occur on experimentally relevant time scales. To capture this, we derive the decomposition energy per atom for each composition from Pourbaix diagrams at the working pH and potential. This decomposition energy directly measures the thermodynamic driving force for electrochemical decomposition into stable oxides/hydroxides/ions in the given electrolyte and is more relevant than using, for example, the entropic preference for a random solid solution. In our multi-objective optimization, we therefore use the negative decomposition energy per atom and the negative overpotential as stability and activity objectives to be maximized. Because catalyst degradation for OER catalysis can be severe at lower potentials, our choice of stability metric allows us to directly compare theory with experiment. Under OER conditions, all catalysts exhibit measurable performance decay within a limited time window. By using Pourbaix-based decomposition energies as a stability axis, we can correlate the predicted thermodynamic driving force for in-electrolyte decomposition with experimentally measured degradation (loss of activity and structural changes) and identify compositions that optimally balance high OER activity with acceptable finite-time stability. This provides an explicit, quantitative connection between multi-objective ML–DFT screening and experimentally observed degradation. With the Pareto front based on physically realistic electrochemical descriptors as a guide, one can strategically balance competing catalyst properties to suit specific operational needs.

Advances in our understanding of catalysis in specific applications might suggest other features, in addition to stability and activity, that must be optimized (for example, selectivity or cost), and the present methods could in principle be extended to multi-objective optimization of three or more features. In this regard, we direct the reader to a machine-learning-density-functional multi-objective optimization framework for HEA oxygen-reduction-reaction (ORR) electrocatalyst (the ORR is a reverse reaction of the OER studied in the present work) that maps Pareto fronts in activity, cost, and mixing entropy as a stability proxy.^64^ The problem setting and overall system perspective in that paper align closely with the present work, such that the studies provide an opportunity to compare computational strategies. In particular, their activity feature is a distribution of *O and *OH adsorption energies on HEA(111) surfaces, whereas ours is involves the four-electron energy landscape at each surface site, and their stability feature is ideal mixing entropy as a measure of thermodynamic driving force to form single-phase solid solutions whereas our stability feature is decomposition energy per atom for each composition from Pourbaix diagrams at the working pH and potential. This illustrates how a variety of features can be used for multi-objective catalyst design.

## Author contributions

Z. W., D. G. T., and Y. Z. supervised the project. C. Z. performed the computational work and plotted the figures. Q. S. collected the experimental data. C. Z. and D. G. T. co-wrote the manuscript. All authors discussed the results and assisted with the manuscript preparation.

## Conflicts of interest

There are no conflicts to declare.

## Supplementary Material

SC-017-D5SC06100H-s001

SC-017-D5SC06100H-s002

## Data Availability

All data supporting the findings of this study are presented in the article and supplementary information (SI). Supplementary information: (1) a PDF file containing flow chart and overview, computational methods and software, descriptors for the OER, details of the genetic algorithm, and stability filtering; (2) a ZIP file containing code and data. See DOI: https://doi.org/10.1039/d5sc06100h.
