# Supplementary material for: Finding the Pareto front for high-entropy-alloy catalysts
Source: Chem Sci. 2026 Jan 9;17(9):4744–52. doi: 10.1039/d5sc06100h (PMC12802164; doi:10.1039/d5sc06100h)
Supplement: SC-017-D5SC06100H-s001 [file SC-017-D5SC06100H-s001.pdf]

Supplementary Information (SI) for Chemical Science.
This journal is © The Royal Society of Chemistry 2026

ELECTRONIC SUPPLEMENTARY INFORMATION

Finding the Pareto front for high-entropy-alloy catalysts

Chengyi Zhang[a], Ruihu Lu[a], Qi Sun[a], Yu Mao[a], Tilo Söhnel[a], Yan Zhao[b,*], Donald G. Truhlar[c,*], Ziyun Wang[a,*]

[a] School of Chemical Sciences, University of Auckland, Auckland 1010, New Zealand

[b] College of Material Science and Engineering, Sichuan University, Chengdu 610065, China

[c] Department of Chemistry, Chemical Theory Center, and Supercomputing Institute, University of Minnesota, 207 Pleasant Street S.E., Minneapolis, MN 55455-0431, USA

* Corresponding authors: Yan Zhao (yanzhao@scu.edu.cn), Donald G. Truhlar (truhlar@umn.edu), and Ziyun Wang (ziyun.wang@auckland.ac.nz).

Table of Contents

S1. Flow chart and overview 3
S2. Calculation methods and software........ 4
S2.1. Site preparation 4
S2.2. Graph neural network model 6
S2.3. Density functional calculations 6
S3. Descriptors for the oxygen evolution reaction (OER) 7
S3.1. Activity descriptor 7
S3.2. Stability descriptor 9
S4. Details of the genetic algorithm 9
S4.1. Assigning Fitness 9
S4.2. Evolving the Next Generation 12
S4.3. Determination of the hyperparameters 13
S4.3.1. Number of offspring per generation and number of generations 13
S4.3.2. Parameters controlling mutation number and niche size 15
S5. Stability filtering 16
References........ 18

S1. Flow chart and overview

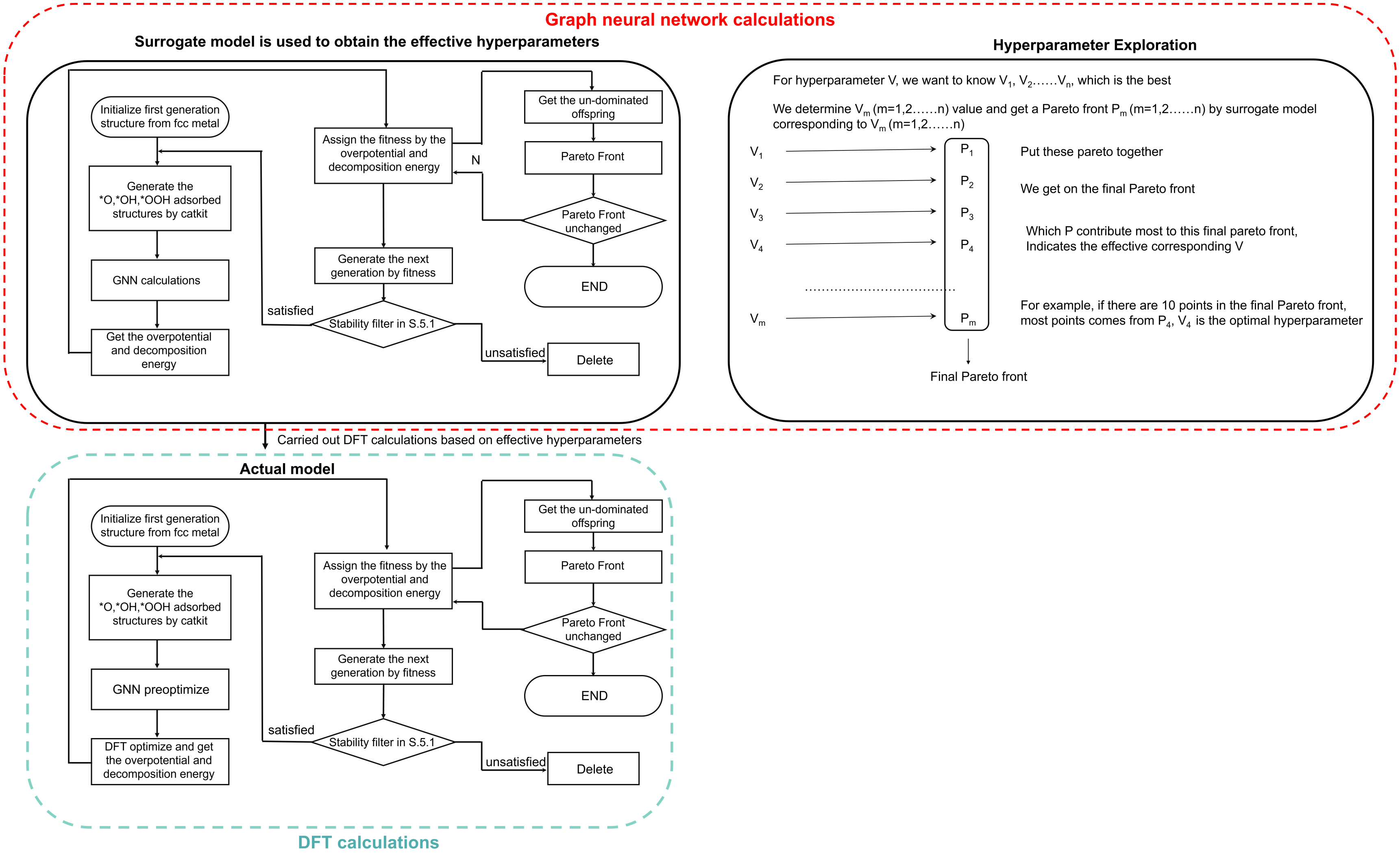

Fig S1. Flow chart of the Pareto front exploration process. GNN denotes graph neural network. DFT denotes density functional theory. Y denotes yes. N denotes no.

In this work, we pose the bi-objective problem as a maximization problem, where the objectives to be maximized are a stability descriptor and an activity descriptor. Fig S1 provides an overview of the optimization process for finding the Pareto front. The top panels of the figure concern the surrogate stage in which graph neural network (GNN) model is used to optimize hyperparameters. Hyperparameters are parameters that affect the rate of learning. The optimized hyperparameters are used in the density functional (DF) stage, given by the bottom panel. Both the surrogate stage and the final DF stage are complete and independent exploration processes. The surrogate stage involves several genetic algorithm optimizations based on GNN structures and energies and is used solely to determine effective hyperparameters, and the final stage (we restart from the first generation of face-centered-cubic (FCC) metals) is a single genetic algorithm optimization using DF structures and energies to obtain the final Pareto front. The workflow of these stages is described in the main text. This SI Appendix gives details.

Throughout the SI Appendix, the HEA catalysts are referred to as materials.

Section 2 provides further details of the site preparation, the GNN model, and the DF calculations.

The stability descriptor is defined as the negative decomposition energy evaluated at 1.23 V vs. RHE and pH = 7, where larger values correspond to higher thermodynamic stability (see Section S3.2 for details).

The activity descriptor is defined as the negative of the calculated overpotential for the oxygen evolution reaction (OER), obtained from the Gibbs free energies of *O, *OH, and *OOH adsorption at 1.23 V versus RHE. The larger value of this descriptor therefore corresponds to higher catalytic activity. Details of the calculation procedure are provided in Section S2.1.

Section 4 gives further details of various aspects of the generic algorithm calculations.

Section 5 gives details of stability filtering and activity screening.

Sample codes are provided in supplementary files.

S2. Calculation methods and software

S2.1. Site preparation

The structures of the initial generation were face-centered-cubic metals taken from the Materials Project.[1] We first generated *O structures on face-centered cubic metals. To regulate the structures, we employed the *Atomic Simulation Environment* (ASE)[2, 3].

Because it is impractical to manually inspect every alloy structure generated during the iterative process of the genetic algorithm, we made several assumptions. We assumed the (111) surfaces of the FCC structures are the most stable facets. Based on previous literature[4, 5], oxygen adsorption is generally more favorable at FCC hollow sites than at top and bridge sites, as presented in Fig.S2. Therefore, we first generated all possible O adsorption configurations using the *CatKit*[6] code. Subsequently, we removed all configurations where O was adsorbed at top or bridge sites to reduce the computational cost; that is, we

considered only the threefold hollow sies for adsorption. Therefore, we first determined the five most stable *O adsorption configurations on each surface. Based on this optimized *O structure, *OH and *OOH species were subsequently placed at the same adsorption site and fully relaxed. The corresponding Gibbs free energies were then used to evaluate the overpotential at each site.

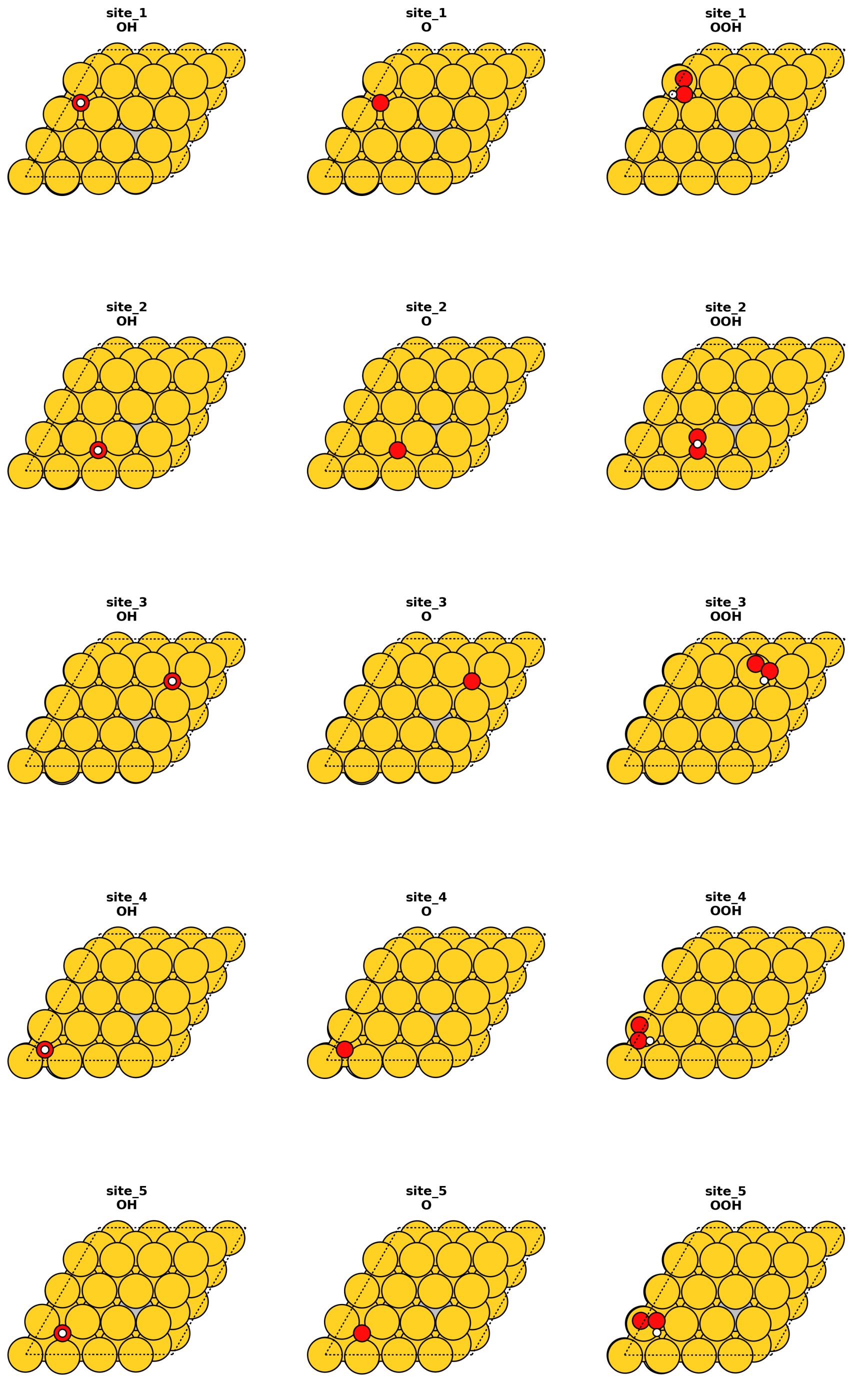

Fig S2. *OH, *O, and *OOH adsorption sites sampling on the (111) surfaces of a certain alloy, for example.

All our initial structures are assumed to be face-centered cubic structures to simplify the model. All oxygen atoms are adsorbed on the (111) surface of the material with five layers using ASE with a vacuum layer of at least 15 Å. The bottom two layers were fixed and defined as those atoms more than 3 Å from the top of the surface.

S2.2. Graph neural network model

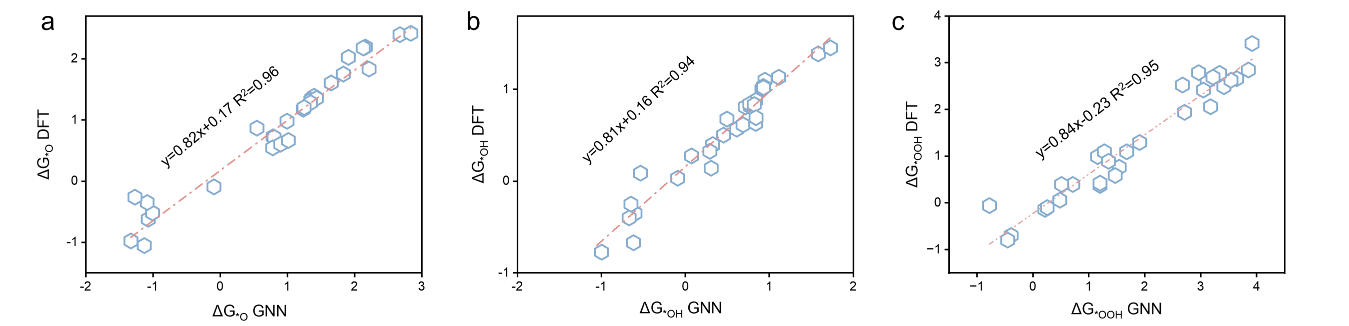

Fig S3. The comparison of the G_{*O} G_{*OH}, and G_{*OOH} by the graph neural network (GNN) and DFT.

UMA-m-1p1 is a universal graph neural network interatomic potential from Meta FAIR's UMA family, pretrained on billions of atomic environments spanning the OC20 heterogeneous catalysis dataset together with ODAC23, OMat24, OMol25, and OMC25, all computed at the DFT level using standardized exchange–correlation functionals and convergence criteria. UMA-m-1p1 (oc20 task in FAIRChemCalculator) was employed as a surrogate model to predict atomic energies and forces and to pre-relax adsorbate–surface structures with near-DFT accuracy prior to final DFT refinements.

S2.3. Density functional calculations

For the final determination of the Pareto front, the structures were first pre-optimized using the GNN surrogate model, followed by DF calculations for refined geometry optimization and accurate energy evaluation.

All DF simulations were performed by Vienna Ab initio Simulation Package (VASP)[7-9] using the PBE-D3(BJ) density functional[10, 11] and the projector augmented wave (PAW) method to account for core-valence interactions.[12] The reciprocal space was sampled by the Γ-centered Monkhorst−Pack scheme with 5 ×5 ×5 *k*-point grids for calculating formation energies of bulk materials and 2 ×2 ×1 *k*-point grids for calculating the adsorption energies. We used an energy cutoff of 450 eV.[13] All density functional calculations were spin-restricted, except for those involving Ni, Fe, and Co, for which spin-polarized calculations were performed.[14] The initial charge density was generated from the superposition of atomic charge densities. Electronic self-consistent field (SCF) iterations were performed using the Fast algorithm, with a convergence criterion of 1×10^{-7} eV for the total energy. The number of SCF steps ranged between 5 and 60. The projection operators were evaluated in real or reciprocal space as determined automatically. Symmetry operations were disabled to allow for full structural flexibility during relaxation.

Geometry optimizations were performed using the conjugate gradient method, with a step scaling factor of 0.2. The atomic positions were relaxed until the maximum force on each atom was below 0.05 eV/Å, with a maximum of 1000 ionic steps allowed. The cell shape and volume were fixed during the relaxation. An implicit solvation model was enabled with VASPSOL [15, 16] with the solvent dielectric parameter set to 80 to approximate aqueous conditions.

S3. Descriptors for the oxygen evolution reaction (OER)

S3.1. Activity descriptor

The OER is intrinsic to many energy technologies, including water splitting, rechargeable metal-air batteries, and fuel cells, but it is a sluggish reaction. The OER is the production of O_2 molecules from water by the following four-electron process:[17]

$$2H_2O \rightarrow O_2 + 4H^+ + 4e^- \quad (S1)$$

The standard-state reduction potential is E^0 = 1.23 V vs. the reversible hydrogen electrode (RHE). The four-step OER process involves three OER intermediates, namely, *OH, *O, and *OOH:

$$H_2O + * \rightarrow OH^* + H^+ + e^- \quad (S2)$$

$$OH^* \rightarrow O^* + H^+ + e^- \quad (S3)$$

$$O^* + H_2O \rightarrow OOH^* + H^+ + e^- \quad (S4)$$

$$OOH^* \rightarrow * + O_2 + H^+ + e^- \quad (S5)$$

where *, OH*, O*, and OOH* represent the adsorption site, absorbed OH, adsorbed O, and adsorbed OOH, respectively. The free energy changes (ΔG_i, i = 1, 2, 3, 4) of the elementary steps are:

$$\Delta G_1 = G_{OH^*} + G_{H^+ + e^-} - G_{H_2O} - G_* \quad (S6)$$

$$\Delta G_2 = G_{O^*} + G_{H^+ + e^-} - G_{OH^*} \quad (S7)$$

$$\Delta G_3 = G_{OOH^*} + G_{H^+ + e^-} - G_{O^*} - G_{H_2O} \quad (S8)$$

$$\Delta G_4 = G_* + G_{O_2} + G_{H^+ + e^-} - G_{OOH^*} \quad (S9)$$

A major goal in the field of OER electrocatalysis has been to find or design an efficient electrocatalyst that has high abundance and low cost. One generally needs a voltage above 1.23 V (such as 1.5 V) to drive OER on specified materials (catalysts) under a specified current density (e.g., 10 mA cm^{-2}), and this overpotential means that one requires extra energy input. Therefore, the overpotential of the drive voltage is an important descriptor. The steady-state approximation gives a lower limit on the overpotential of:

$$E_{\text{overpotential}} \geq \max(\Delta G_1, \Delta G_2, \Delta G_3, \Delta G_4)/e - E^0 \quad (S10)$$

The energy changes of the elementary steps are related to the adsorption behavior of OER intermediates. We consider the formation energies of the OER intermediates relative to H_2O and H_2

molecules:

$$2H_2O + * \rightarrow OOH^* + 3/2H_2 \tag{S11}$$

$$H_2O + * \rightarrow OH^* + 1/2H_2 \tag{S12}$$

$$H_2O + * \rightarrow O^* + H_2 \tag{S13}$$

With this convention, the corresponding adsorption energies of OER intermediates can be calculated as follows:

$$\Delta G_{OOH^*} = G_{OOH^*} + 3/2G_{H_2} - 2G_{H_2O} - G_* \tag{S14}$$

$$\Delta G_{OH^*} = G_{OH^*} + 1/2G_{H_2} - G_{H_2O} - G_* \tag{S15}$$

$$\Delta G_{O^*} = G_{O^*} + G_{H_2} - G_{H_2O} - G_* \tag{S16}$$

and the free energy changes of element steps and be calculated as follows:

$$\Delta G_1 = \Delta G_{OH^*} \tag{S17}$$

$$\Delta G_2 = \Delta G_{O^*} - \Delta G_{OH^*} \tag{S18}$$

$$\Delta G_3 = \Delta G_{OOH^*} - \Delta G_1 - \Delta G_2 \tag{S19}$$

$$\Delta G_4 = 4.92 - \Delta i_{OOH^*} \tag{S20}$$

Equations S10 and S14-S21, taken together, provide a relation between the overpotential and the absorption free energy.

We define the activity descriptor as follows:

$$Z_a = -|\mathrm{Max}(\Delta G_1, \Delta G_2, \Delta G_3, \Delta G_4) - 1.23 \text{ eV}| \tag{S21}$$

For each reaction energy on the HEAs, we explicitly enumerated all symmetry-inequivalent threefold hollow sites defined by the local metal triatomic hollow sites on the surface. For each such site, we first optimized the adsorption geometry of *O by placing an oxygen atom above the hollow and allowing full structural relaxation. The optimized *O configuration at a given hollow was then used as a common lateral template for the other intermediates: *OH and *OOH was initially placed such that the oxygen atom occupied the same threefold hollow position as *O, with the H atom oriented away from the surface, and the adsorbate was subsequently relaxed without constraints. In this way, *OH and OOH* were allowed to migrate during optimization to their locally preferred configurations in the vicinity. For every optimized intermediate (*O, *OH, *OOH) at each point, we evaluated the Gibbs free energies using the computational hydrogen electrode framework and constructed the complete four-step OER free-energy profile.

With the assumption that the greater the deviation, the poorer the materials' activity, whether due to excessively strong or weak adsorption. Here, we calculate the overpotential of all sites and choose the minimum overpotential as the activity of the metal. Note that we pose the bi-objective problem as a maximization problem, where one of the objectives to be maximized is activity; maximizing the negative

number of equation S21 gives a less negative number and hence a smaller absolute value of the deviation from 0; therefore, maximizing the descriptor maximizes the calculated activity.

S3.2. Stability descriptor

We define the stability descriptor Z_s as decomposition energy per atom at 1.23V vs. RHE and pH of 7 derived by the ASE

$$\Delta G_{\text{decomp}}(U, \text{pH}) = G_{\text{phase}}(U, \text{pH}) - \min_j \left[\sum_i n_{ij}\mu_i(U, \text{pH})\right] \quad \text{(S22)}$$

$\Delta G_{\text{decomp}}(U, \text{pH})$ denotes the Gibbs free energy per atom of the target material (such as the alloy or solid phase) under a given potential $U$and pH. The term $\mu_i(U, \text{pH})$represents the electrochemical chemical potential of species i(for example, M^{2+}, $M(OH)_3$, H_2O, or O_2) at the same conditions. The subscript $j$indexes all possible decomposition reactions, corresponding to combinations of stable phases that define the lower-energy envelope of the Pourbaix diagram. The stoichiometric coefficient n_{ij}specifies the number of units of species i involved in the decomposition pathway j.

In theoretical exploration, we assessed activity based on the negative value of deviates from the optimal 0 eV, i.e., larger deviations indicate higher activity. Accordingly, on the activity-stability plots, a larger abscissa value corresponds to better activity. When comparing experimental results with theoretical predictions, we use the reciprocal of the overpotential: a larger overpotential leads to a smaller reciprocal, aligning with the computational perspective that a smaller abscissa value represents better activity. Meanwhile, for stability, a more positive cohesive energy per atom (i.e., a lower ordinate value) indicates greater stability. The positive value indicates that the materials are more stable than their reference states.

To address the issue caused by the different units (dimensions) of activity and stability, we normalized the differences in activity and stability in equation S25 by dividing each term by its respective range ($Z_s^{max} - Z_s^{min}$ and $Z_a^{max} - Z_a^{min}$). This eliminates bias arising from differences in dimensional scales.

S4. Details of the genetic algorithm

S4.1. Assigning Fitness

We use the concept of Pareto dominance in evaluating fitness and assigning selection probability. The materials in the population are ranked according to a dominance rule, and each material is assigned a fitness value based on its rank. We first rank all materials in generation t:

$$r(x, t) = 1 + \sum_{y \in P, y > x} r(y, t) \quad \text{(S23)}$$

where $r(x, t)$ is the number of materials dominating material x at generation t, and the restriction $y > x$

means that x is dominated by y (i.e., that material y has better stability and better activity than x). Equation S.23 is illustrated in Fig.3. The a, b, c, and d points in the figure are non-dominated materials; the r value of these points is $r = 1 + 0 = 1$. Point e is dominated by point a, which has an r value of 1; therefore, the r value of e is $r = 1 + 1 = 2$. Point h is dominated by points a, b, and f, giving it an r value of $r = 1 + 1 + 1 + 2 = 5$.

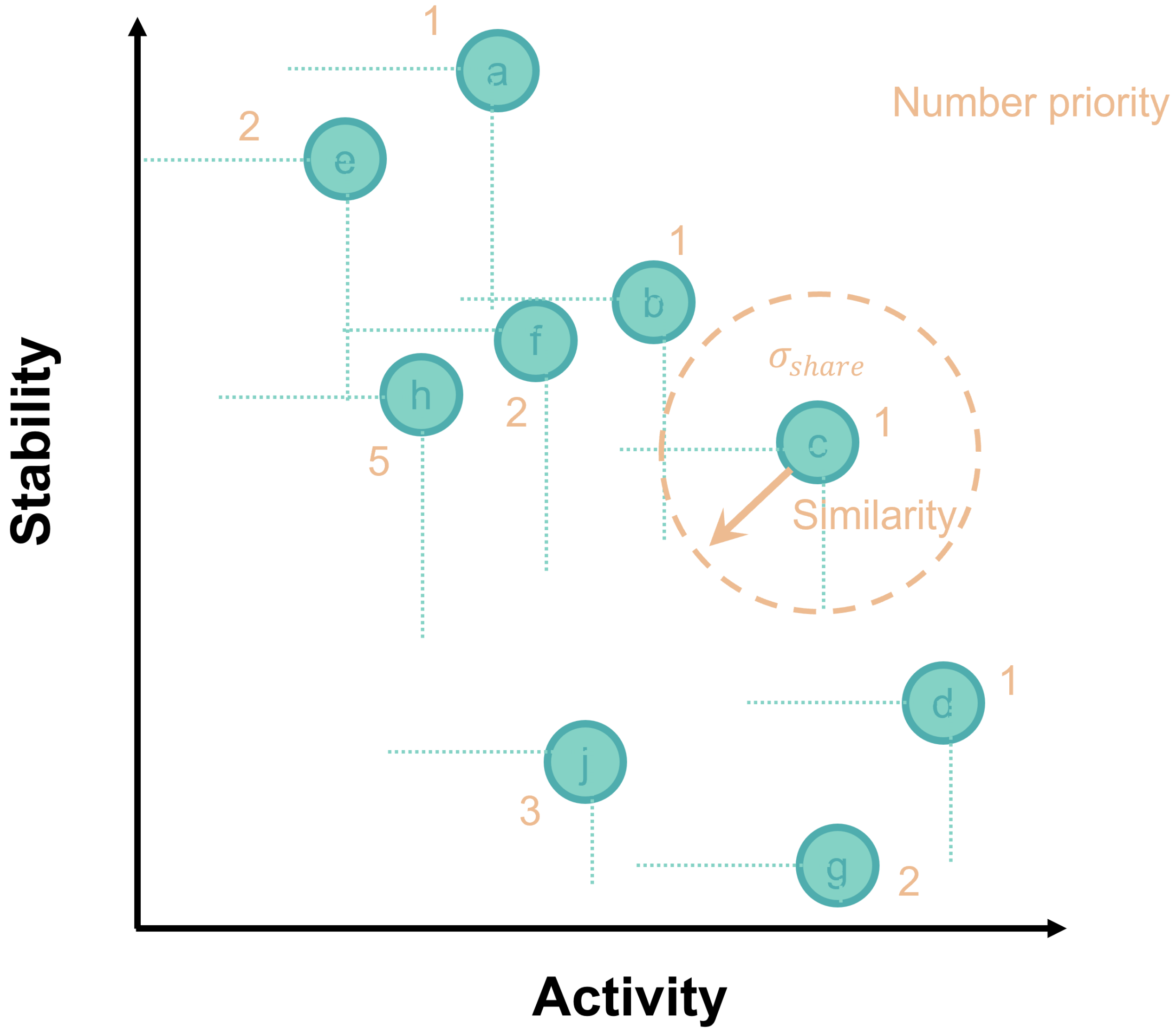

Fig S4. A sample set of materials, where the materials are HEA catalysts. The text following equation S23 uses this diagram to illustrate the assignment of r values. The diagram also illustrates the niche size σ_{share}, which is the niche size and which gives information about the similarity of the different materials. The niche size is associated with the radius centered on a specific HEA candidate, and it is discussed below equation S24.

The next step is to assign fitness values to each material based on the material's rank. The fitness is

initially defined by

$$f(x,t) = N - \sum_{k=1}^{r(\mathbf{x},t)-1} n_k - 0.5\left(n_{r(\mathbf{x},t)} - 1\right) \tag{S24}$$

where n_k is the number of materials with rank *k*. Then, we define the Euclidean distance $\Delta z(x,y)$ between every material pair *x* and *y* as follows

$$\Delta z(x,y) = \sqrt{\left(\frac{Z_s(x) - Z_s(y)}{Z_s^{max} - Z_s^{min}}\right)^2 + \left(\frac{Z_a(x) - Z_a(y)}{Z_a^{max} - Z_a^{min}}\right)^2} \tag{S25}$$

where Z_s and Z_a denote respectively the value of the stability descriptor and the value of the activity descriptor, and we use these distances to calculate a niche count defined as follows:

$$nc(x,t) = \sum_{y \in P, r(\mathbf{y},t) = r(\mathbf{x},t)} \max\left\{\frac{\sigma_{\text{share}} - \Delta z(x,y)}{\sigma_{\text{share}}}, 0\right\} \tag{S26}$$

where the condition $y \in P$ in equation S26 indicates that *y* is another material in generation *P* that we are comparing to x, and σ_{share} is the niche size, which is a hyperparameter determined in the surrogate stage. The circle around of radius σ_{share} around a given material in property space encloses a niche, and if many offspring fall within this niche, the fitness of that central candidate is penalized accordingly. This mechanism discourages the search algorithm from generating offspring that cluster near one another by penalizing materials in overcrowded regions. This promotes diversity in the population and encourages broader exploration of the design space by increasing the likelihood of discovering a well-distributed set of globally competitive candidates and ultimately of identifying a more representative and complete Pareto front. After calculating niche counts by means of equation S26, the fitness of each material is adjusted as follows:

$$f^{\text{adj}}(x,t) = \left[f^{\text{norm}}(x,t) n_{r(\text{x},t)} f(x,t)\right] \Big/ \sum_{y \in P, r(\mathbf{y},t) = r(\mathbf{x},t)} f^{\text{norm}}(x,t) \tag{S27}$$

where

$$f^{\text{norm}}(x,t) = \frac{f(x,\text{t})}{n_c(x,t)} \tag{S28}$$

Then we normalized the adjusted fitness of all materials in this generation to the range [0,1]. This algorithm considers the similarity of the materials to avoid the search for the Pareto front being trapped in a local maximum.

The fitness of each material controls the probability that the material is chosen to generate offspring.

S4.2. Evolving the Next Generation

Each time an offspring is to be generated, we first pick a random number in the range [0,1]. This number is compared with the fitness of a randomly selected parent. If the fitness of the selected parent is greater than the random number, the offspring is generated directly; otherwise, another parent is selected. This ensures that individuals with higher fitness have a greater probability of being preserved.

Then we determine whether the offspring will be generated through the mutation algorithm or the crossover algorithm. Specifically, for each new offspring to be generated, we first draw a random number of 0 or 1. Based on this random value, we randomly select whether the offspring will be created via the mutation algorithm or the crossover algorithm. Note that the mutation algorithm always leads to mutation, but the "crossover algorithm" may lead to either mutation or crossover.

Mutation algorithm. If mutation was chosen, we randomly select a material. If the fitness of the selected material is lower than the random number, we continue selecting new materials until one with a fitness higher than the random number is found. When the fitness of that material is greater than the random number, we perform a mutation. For this purpose, we define a parameter called the mutation number. The mutation number is the number of atoms randomly selected (from the 32 atoms that constitute our bulk structure) that are replaced by atoms of different elements during the mutation step. For example, a mutation number of 4 means that 4 atoms in the bulk structure are randomly chosen and substituted with different elements. This parameter allows us to systematically explore a wide range of structural and compositional variations. As explained on Section S4.3.3, we finally selected mutation number = 1.

Crossover algorithm. The crossover operation generates offspring structures by implementing a spatial cut-and-splice mechanism: first, one of the three crystallographic axes (a, b, or c) is randomly selected as the cutting direction; second, a cutting position along this axis is determined by sampling a ratio parameter percent_2 from a Beta distribution, where 70% of the time a Beta(3.0, 3.0) distribution (scaled to range 0.3-0.7) is used to favor balanced cuts near the midpoint, and 30% of the time a Beta(1.2, 1.2) distribution (scaled to range 0.1-0.9) is used to allow more aggressive recombination—additionally, when the fitness difference between parents exceeds 0.25, a bias of up to 0.05 is added toward the higher-fitness parent to preserve superior genetic material; third, atoms from Parent 1 with coordinates below the cut position along the selected axis are retained while atoms from Parent 2 with coordinates at or above the cut position are retained; fourth, these two complementary atom sets are combined to form the offspring, which inherits the cell parameters from Parent 1, and the spatial partitioning naturally maintains the fixed atom count of 32 since approximately (1-percent_2)×32 atoms from Parent 1 and percent_2×32 atoms from Parent 2 are combined; finally, the offspring undergoes quality validation

where interatomic distances are checked against 0.6 times the sum of covalent radii, and if conflicts are detected, small random perturbations (±0.1 Å) are applied to resolve them—this entire process is attempted up to 50 times with different cutting positions, and if all attempts fail to produce a valid offspring, the algorithm falls back to mutation of the higher-fitness parent to ensure continuous population evolution.

S4.3. Determination of the hyperparameters

We used the surrogate model to identify the values of several hyperparameters that can accelerate the search for the Pareto front. The three hyperparameters chosen are the cutoff parameters for the number of offspring per generation, the number of generations, the mutation number, and the niche size. Although we aimed to identify combinations that effectively balance search efficiency and convergence, the hyperparameters were selected based on limited trial-and-error, and we do not claim to have found the absolutely optimal values. Furthermore, it is well known that the optimum values of hyperparameters depend on the problem under study [18-21]. However, it is also well known that some multi-objective approaches are better than others [22, 23]. We believe that our surrogate model is sufficiently close to the final DF model, making the determination of hyperparameters in the surrogate stage useful for improving efficiency.

S4.3.1. Number of offspring per generation and number of generations

We analyzed the effect of offspring number per generation on Pareto front exploration efficiency under fixed computational cost constraints. To mitigate the computational cost, we maintained a constant total number of evaluated structures (600) across all tests. We define O as the number of offspring per generation and G as the number of generations. Thus, the product of O and G for each O-G pair is 600. Varying O with this constraint involves a trade-off between search breadth (number of offspring per generation) and search depth (number of generations). For each O-G pair, the GA was executed independently, and all generated structures were collected, as shown in Fig.S5. We then constructed a unified final Pareto front by combining the Pareto fronts of each O-G pair. The contribution of each pair to the final Pareto front was determined by counting the number of its generated structures that lie on it. This reflects the ability of each configuration to discover high-performing structures under the same computational budget. In Fig.S6, a higher bar indicates greater efficiency in discovering globally optimal trade-offs between catalytic activity and stability under the same computational cost. Fig. S6 shows that the Pareto front generated with $G = 15$ and $O = 40$ contributes the most points to the global Pareto front; therefore, this pair is considered the most effective and was selected as our optimized pair for use in the second stage.

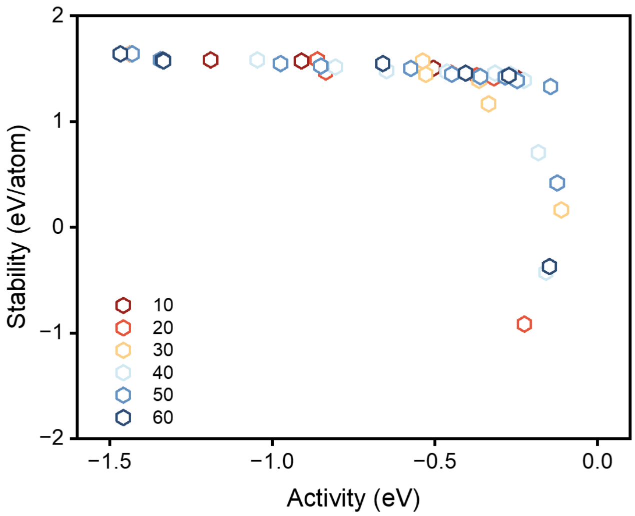

Fig S5. The contribution of each O-G pair to the final Pareto front. The legend gives the offspring count O and the product of the offspring count, and the number of generations P is fixed at 600. Thus, the figure includes the following O-G pairs: 10-60, 20-30, 30-20, 40-15, 50-12, and 60-10.

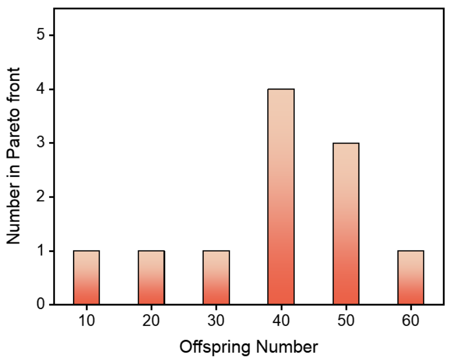

Fig S6. The number of structures from each *O–G* pair that appear on the final unified Pareto front. Each offspring–generation pair has a fixed total of 600 structures. Each bar represents the number of structures that contribute to the final Pareto front constructed from all runs.

S4.3.2. Parameters controlling mutation number and niche size

Similarly, we used the surrogate model to optimize the other hyperparameters, which are the mutation number and the σ_{share} value. The explanation of the mutation number is in Section S4.2. The explanation of σ_{share} is in Section S4.1. The results are summarized in Figs S7–S9.

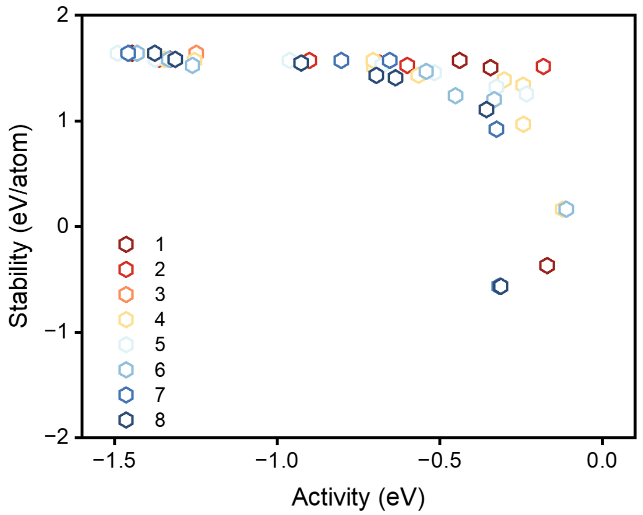

Fig S7. The contribution of each mutation number to the final Pareto front.

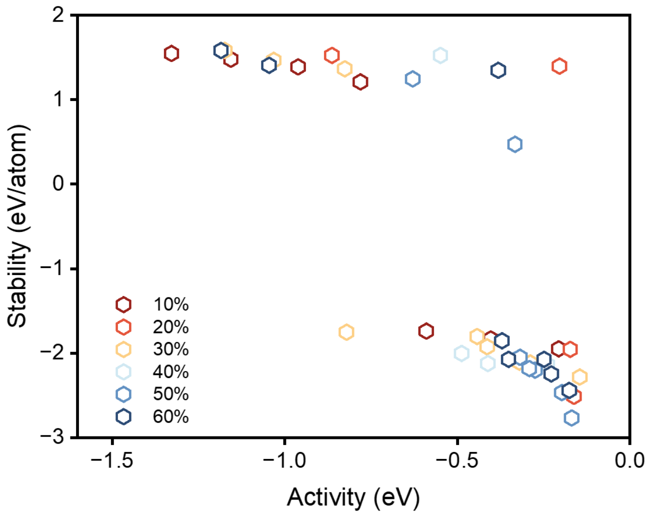

Fig S8. The contribution of each σ_{share} value to the final Pareto front.

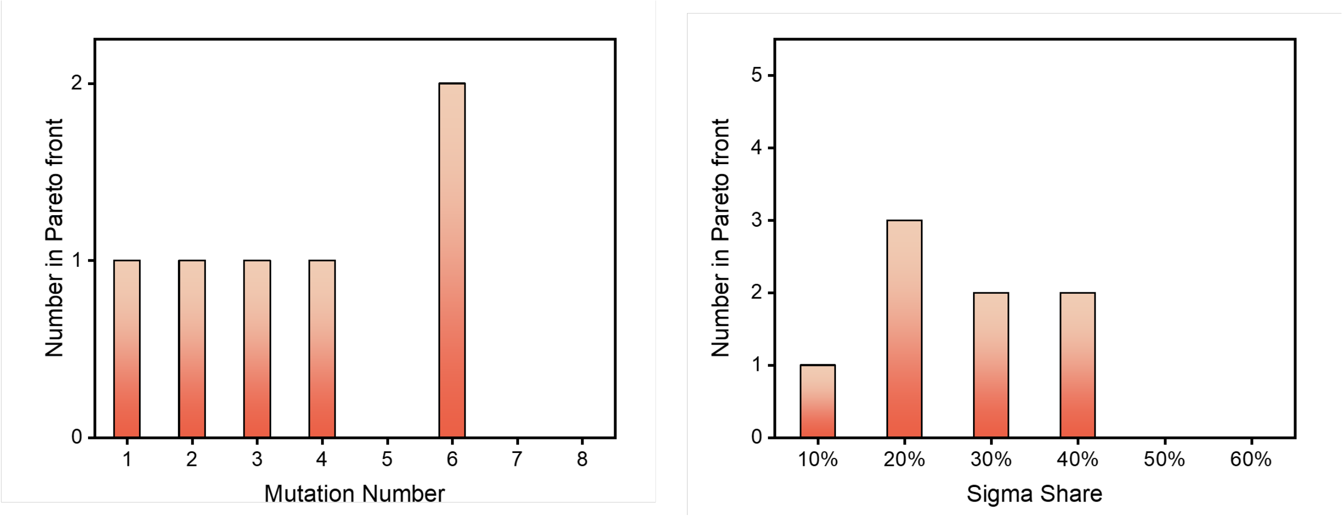

Fig S9. Summary of parameter optimization results for the genetic algorithm, showing the number of structures from each parameter setting that appear on the final unified Pareto front. The figure combines two independent tests: (a) mutation number performance under the optimal offspring configuration (40 offspring × 15 generations), and (b) σ_{share} values applied with mutation number = 6 to enhance search diversity. Each bar represents the number of structures generated under the corresponding parameter setting that contributes to the final Pareto front constructed from all runs. A higher bar indicates greater efficiency in discovering globally optimal trade-offs between catalytic activity and stability under the same computational cost.

S5. Stability filtering

In both stages, we employed an empirical formula method (details below) for filtering of material-phase stability.

The goal of stability filtering is to rule out materials where the metals of the alloy phase separate rather than mixing [24, 25]. We adopt the three parameters of the constituent elements in the multi-component alloys to characterize the phase stability of the materials. The parameters, taken from previous work [26, 27], are the atomic size difference (δ), the mixing enthalpy (ΔH_{mix}), and the mixing entropy (ΔS_{mix}). They are defined by

$$\delta = 100\sqrt{\sum_{i=1}^{n} c_i\left(1-\frac{r_i}{r_a}\right)^2} \tag{S32}$$

where

$$r_a = \sum_{i=1}^{n} c_i r_i \tag{S33}$$

c_i is the atomic percentage and atomic radium of the *i*th element, r_i is the atomic radius of the *i*th element, and the numerical factor 100 was used for convenience;

$$\Delta H_{\text{mix}} = \sum_{i=1, i\neq j}^{n} \Omega_{ij} c_i c_j \tag{S34}$$

where

$$\Omega_{ij} = 4\Delta_{\text{mix}}^{AB}, \Delta_{\text{mix}}^{AB} \tag{S35}$$

is the mixing enthalpy of the binary liquid alloy AB; and

$$\Delta S_{\text{mix}} = -R \sum_{i=1}^{n} c_i \ln c_i \tag{S36}$$

where R is the gas constant. We employed the criteria $0 \leq \delta \leq 8.5$, $-22 \leq \Delta H_{\text{mix}} \leq 7$ kJ/mol, and $11 \leq \Delta S_{\text{mix}} \leq 19.5$ J $K^{-1} \cdot mol^{-1}$. These criteria are accepted in the literature to determine whether the components can form high-entropy alloys instead of segregating.[28-30]

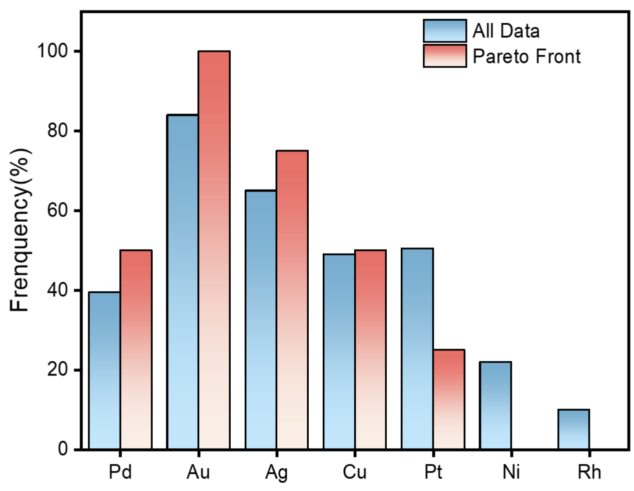

Fig S10. The top element frequency in all data and on the Pareto front.

References

1. A. Jain, S. P. Ong, G. Hautier, W. Chen, W. D. Richards, S. Dacek, S. Cholia, D. Gunter, D. Skinner, G. Ceder and K. A. Persson, Commentary: The Materials Project: A materials genome approach to accelerating materials innovation, *Apl. Mater.*, 2013, **1**.
2. A. Hjorth Larsen, J. Jorgen Mortensen, J. Blomqvist, I. E. Castelli, R. Christensen, M. Dulak, J. Friis, M. N. Groves, B. Hammer, C. Hargus, E. D. Hermes, P. C. Jennings, P. Bjerre Jensen, J. Kermode, J. R. Kitchin, E. Leonhard Kolsbjerg, J. Kubal, K. Kaasbjerg, S. Lysgaard, J. Bergmann Maronsson, T. Maxson, T. Olsen, L. Pastewka, A. Peterson, C. Rostgaard, J. Schiotz, O. Schutt, M. Strange, K. S. Thygesen, T. Vegge, L. Vilhelmsen, M. Walter, Z. Zeng and K. W. Jacobsen, The atomic simulation environment-a Python library for working with atoms, *J. Phys.: Condens. Matter*, 2017, **29**, 273002.
3. S. R. Bahn and K. W. Jacobsen, An object-oriented scripting interface to a legacy electronic structure code, *Comput. Sci. Eng.*, 2002, **4**, 56-66.
4. Y. Sun and S. Dai, High-entropy materials for catalysis: A new frontier, *Sci. Adv.*, 2021, **7**.
5. W. Xu, E. Diesen, T. He, K. Reuter and J. T. Margraf, Discovering High Entropy Alloy Electrocatalysts in Vast Composition Spaces with Multiobjective Optimization, *J. Am. Chem. Soc.*, 2024, **146**, 7698-7707.
6. J. R. Boes, O. Mamun, K. Winther and T. Bligaard, Graph Theory Approach to High-Throughput Surface Adsorption Structure Generation, *J. Phys. Chem. A*, 2019, **123**, 2281-2285.
7. G. Kresse and J. Hafner, Ab initio molecular dynamics for liquid metals, *Phys. Rev. B: Condens. Matter*, 1993, **47**, 558-561.
8. G. Kresse and J. Furthmuller, Efficient iterative schemes for ab initio total-energy calculations using a plane-wave basis set, *Phys. Rev. B: Condens. Matter*, 1996, **54**, 11169-11186.
9. G. Kresse and D. Joubert, From ultrasoft pseudopotentials to the projector augmented-wave method, *Phys. Rev. B: Condens. Matter*, 1999, **59**, 1758-1775.
10. S. Grimme, J. Antony, S. Ehrlich and H. Krieg, A consistent and accurate ab initio parametrization of density functional dispersion correction (DFT-D) for the 94 elements H-Pu, *J. Chem. Phys.*, 2010, **132**, 154104.
11. J. P. Perdew, K. Burke and M. Ernzerhof, Generalized Gradient Approximation Made Simple, *Phys. Rev. Lett.*, 1996, **77**, 3865-3868.
12. P. E. Blochl, Projector augmented-wave method, *Phys. Rev. B: Condens. Matter*, 1994, **50**, 17953-17979.
13. H. J. Monkhorst and J. D. Pack, Special points for Brillouin-zone integrations, *Phys. Rev. B*, 1976, **13**, 5188-5192.
14. E. Skulason, T. Bligaard, S. Gudmundsdottir, F. Studt, J. Rossmeisl, F. Abild-Pedersen, T. Vegge, H. Jonsson and J. K. Norskov, A theoretical evaluation of possible transition metal electro-catalysts for N_2 reduction, *Phys. Chem. Chem. Phys.*, 2012, **14**, 1235-1245.
15. K. Mathew, R. Sundararaman, K. Letchworth-Weaver, T. A. Arias and R. G. Hennig, Implicit solvation model for density-functional study of nanocrystal surfaces and reaction pathways, *J. Chem. Phys.*, 2014, **140**, 084106.
16. K. Mathew, V. S. C. Kolluru, S. Mula, S. N. Steinmann and R. G. Hennig, Implicit self-consistent electrolyte model in plane-wave density-functional theory, *J. Chem. Phys.*, 2019, **151**, 234101.
17. J. K. Nørskov, J. Rossmeisl, A. Logadottir, L. Lindqvist, J. R. Kitchin, T. Bligaard and H. Jónsson, Origin of the Overpotential for Oxygen Reduction at a Fuel-Cell Cathode, *J. Phys. Chem. B*, 2004, **108**, 17886-17892.
18. e. G. Papa, *Advances in Evolutionary Algorithms Research*, NY, Nova Science Pub: Hauppauge, 2015.
19. G. J. Koehler, New directions in genetic algorithm theory, *Annals of Operations Research*, 1997, **75**, 49-68.
20. D. H. Wolpert and W. G. Macready, No free lunch theorems for optimization, *IEEE Transactions on Evolutionary Computation*, 1997, **1**, 67-82.

21. S. Droste, T. Jansen and I. Wegener, Optimization with randomized search heuristics—the (A)NFL theorem, realistic scenarios, and difficult functions, *Theoretical Computer Science*, 2002, **287**, 131-144.
22. L. Haiming and G. G. Yen, Rank-density-based multiobjective genetic algorithm and benchmark test function study, *IEEE Transactions on Evolutionary Computation*, 2003, **7**, 325-343.
23. D. a. K. Corne, J., Some multiobjective optimizers are better than others, 2003, DOI: 10.1109/CEC.2003.1299403.
24. L. Fattaruso, When unmixable metals mix, *Phys. Today*, 2024, **2024**.
25. M. C. Troparevsky, J. R. Morris, P. R. C. Kent, A. R. Lupini and G. M. Stocks, Criteria for Predicting the Formation of Single-Phase High-Entropy Alloys, *Phys. Rev. X*, 2015, **5**.
26. A. Takeuchi and A. Inoue, Classification of Bulk Metallic Glasses by Atomic Size Difference, Heat of Mixing and Period of Constituent Elements and Its Application to Characterization of the Main Alloying Element, *Mater. Trans.*, 2005, **46**, 2817-2829.
27. Y. Zhang, Y. J. Zhou, J. P. Lin, G. L. Chen and P. K. Liaw, Solid-Solution Phase Formation Rules for Multi-component Alloys, *Adv. Eng. Mater.*, 2008, **10**, 534-538.
28. K. Wang, Z. X. Liu, L. Liu, T. T. Wang, K. Chen, R. H. Yao, R. Q. Hang and X. H. Yao, Dual-sized macro-micro porous biodegradable Zn-Mg-β-TCP scaffolds for bone defect repair: In *vitro* and in *vivo* evaluation, *J. Mater. Sci. Technol.*, 2026, **242**, 317-329.
29. I. Bencherifa, I. Belkhettab, O. Dabou, K. Derkaoui, A. Chetoui, A. Manseri, B. Hamdoud and M. Saâd, Facile hydrothermal synthesis of novel $Cu_{0.2}Co_{0.2}Zn_{0.2}Mn_{0.2}X$ (X = $Ni_{0.2}$, $Fe_{0.2}$, $Ni_{0.2}Fe_{0.2}$, $Ni_{0.1}Fe_{0.1}$) high-entropy alloy nanoparticles with tunable magnetic properties, *Mater. Sci. Eng. B*, 2025, **321**.
30. K. Xiong, J. Li, K. Zhang, Q. Zhou, L. Huang, X. Li, X. Luo, Z. Ni, Y. Liu, H. Zhu, Q. Zhang and W. Feng, $Al_{0.5}$CoCrFeNi-yNb high-entropy alloys via spark plasma sintering: Microstructural evolution, property enhancement, and wear resistance optimization, *Intermetallics*, 2025, **185**.
